# THE FREQUENCY OF RAW VEGETABLE AND FRUIT CONSUMPTION AND ASSOCIATES WITH DEPRESSIVE MOOD IN JAPANESE OLDER ADULTS

**DOI:** 10.1093/geroni/igae098.4086

**Published:** 2024-12-31

**Authors:** Mai Takase, Yuri Yokoyama, Yu Nofuji, Takumi Abe, Kumiko Nonaka, Hiroshi Murayama

**Affiliations:** Tokyo Metropolitan Institute for Geriatrics and Gerontology, Tokyo, Japan; Tokyo Metropolitan Institute for Geriatrics and Gerontology, Tokyo, Japan; Tokyo Metropolitan Institute for Geriatrics and Gerontology, Tokyo, Japan; Meiji University, Tokyo, Japan; Tokyo Metropolitan Institute for Geriatrics and Gerontology, Tokyo, Japan; Tokyo Metropolitan Institute for Geriatrics and Gerontology, Tokyo, Japan

## Abstract

The consumption of vegetables and fruits are known to associate with better health. It is also proven that the intake of raw and unprocessed vegetables and fruits relate to better mental state in younger adults, due to retainment of micronutrients that could be lost from cooking. However, little is known about the relationship between intake of raw vegetables and fruits and mental state in older adults. In this research, we aimed to clarify the association between the frequency of raw vegetable and fruit consumption and depressive mood in Japanese older adults. A questionnaire survey was conducted at the Wako Cohort Study, which targets community dwelling older adults (≥65 years old) living in Saitama prefecture, Japan (September 2023, N=1,004). The frequency of raw vegetable and fruit consumption (every day vs. less than every day) was used as independent variable, and Geriatrics Depression Scale 5 (score of 2 and above as depressive mood vs. 1 and below as none) was used as dependent variable. The percentage of those who consumed raw vegetable and fruit everyday was 27.2% (n=273) and the percentage of those with depressive mood was 27.4% (n=275). Adjusted with age, sex, marital status, education, self-rated health, number of teeth, comorbidity, BMI, cohabitant, income status, and dietary variation score, a binary regression analysis (n=896) revealed that frequency of raw vegetable and fruit consumption negatively associated with depressive mood (OR=0.62, 95%CI=0.40-0.97). This result suggests the importance of frequent raw vegetable and fruit consumption for better mental state in older adults.

